# Micro-sub regional synapse weakening by mimicking the hyperphosphorylation of microtubule associated protein Tau in dendritic spines

**DOI:** 10.1093/braincomms/fcaf234

**Published:** 2025-06-11

**Authors:** Scott J Mitchell, Seung-Chan Kim, Chotchanit Sunrat, Saviana A Barbati, Keshvi Shah, Ambra Annibali, Kwangwook Cho

**Affiliations:** Department of Basic and Clinical Neuroscience, Institute of Psychiatry, Psychology and Neuroscience, King’s College London, London SE5 9TX, UK; Department of Basic and Clinical Neuroscience, Institute of Psychiatry, Psychology and Neuroscience, King’s College London, London SE5 9TX, UK; Department of Basic and Clinical Neuroscience, Institute of Psychiatry, Psychology and Neuroscience, King’s College London, London SE5 9TX, UK; Department of Basic and Clinical Neuroscience, Institute of Psychiatry, Psychology and Neuroscience, King’s College London, London SE5 9TX, UK; Department of Basic and Clinical Neuroscience, Institute of Psychiatry, Psychology and Neuroscience, King’s College London, London SE5 9TX, UK; Department of Basic and Clinical Neuroscience, Institute of Psychiatry, Psychology and Neuroscience, King’s College London, London SE5 9TX, UK; Department of Basic and Clinical Neuroscience, Institute of Psychiatry, Psychology and Neuroscience, King’s College London, London SE5 9TX, UK

**Keywords:** Tau, phosphorylation, synapse, PACSIN1, dendritic segmentation

## Abstract

The role of microtubule associated protein Tau (Tau) in synaptic function is critical, yet many aspects remain unknown. However, increasing levels of tau phosphorylation has implications for physiological and pathophysiological plasticity. Utilizing human full-length (2N4R) phosphomimic Tau transfection in organotypic hippocampal slice culture, we revealed a regional specificity of synapse dysfunction in dendrites of Cornu Ammonis 1 (CA1) neurons. Specifically, phosphorylation mimic at S396/404 (Tau-PHF1E), a site important for pathophysiology, selectively weakened synapses in the distal portion of CA1 secondary apical dendrites within stratum radiatum, while the proximal region of the same dendrites remained unaltered. Furthermore, in the distal region, the expression of TauPHF1E impaired postsynaptic density-95 expression and dysregulated synaptic plasticity. This phenomenon was contingent on the presence of a key Tau-interactome and AMPA-receptor endocytosis-associated protein; PACSIN1. These findings illustrate that the posttranslational modification of Tau can play a key role in synapse weakening and further implicate the importance of the Tau-interactome PACSIN1 as a pivotal mediated in the process, which with further investigation could open new insights into Tau-associated pathophysiology.

## Teaser

Mitchell *et al*. report that phosphorylation-mimic Tau (TauPHF1E) selectively weakens synapses in distal CA1 dendrites, impairing PSD-95 expression, dynamics and synaptic plasticity. This effect depends on PACSIN1, a key Tau-interacting protein. Collectively highlighting that pTau-mediated synapse weakening is not uniform and the importance of the Tau interactome.

## Graphical Abstract

**Graphical Abstract:**
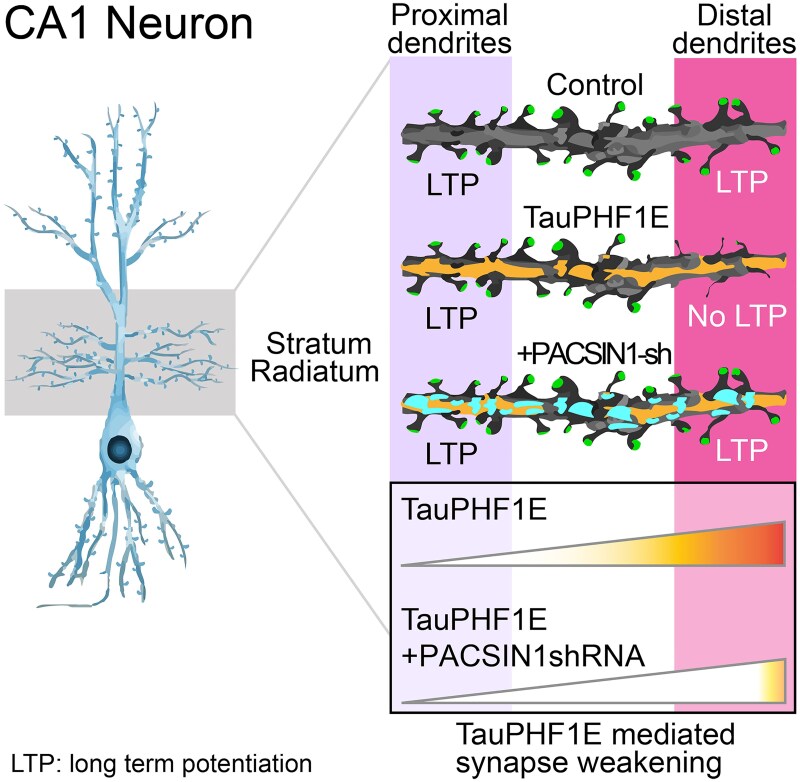

## Introduction

Dendritic microtubule associated protein tau (Tau) has important roles in postsynaptic function and long-term synaptic plasticity.^1,2^ Tau phosphorylation at the residue 396, phosphorylated by GSK3β,^3^ has been shown to be essential for physiological long-term depression (LTD) at the hippocampal synapse.^2,3^ This supports the notion that GSK-3β is one of the key LTD-signalling cascades.^4^ Furthermore, suggesting that GSK-3β-mediated Tau phosphorylation is a molecular mechanism of synapse weakening.^5^

Interestingly, the aberrant phosphorylation of Tau is a phenotype of many neurodegenerative disorders, with phosphorylation levels shown to be four times higher in post-mortem Alzheimer’s disease brains^6^ and suggested to be a causal pathological feature of cognitive impairment, as the phosphorylation of Tau (pTau) has been illustrated to play important roles in homoeostatic plasticity.^2,3,5,7^ However, how this pathophysiology progresses is unknown, as such the progression of pathophysiology and pathology has often been considered from macro levels such as brain region to brain region or functional groups of neurons to neuronal atrophy. However, evidence is beginning to reveal that neurons often exhibit a segmentation that is essential to their function but may additionally underpin how pathophysiology progresses.^8,9^ Interestingly, in senile dementia of Alzheimer’s type, patients’ granular cell distal dendrites exhibit a significant reduction in spine density, while conversely not showing a reduction in the most proximal part of the same dendrites.^10^ More recently, in a model of a-Thalassemia X-linked intellectual disability (ATR-X) syndrome, long-term plasticity in distal apical dendrites was significantly impaired while proximal apical dendrites only exhibited a marginal impairment.^11^ In the tau transgenic model (tauP301L) 4510, the hyperphosphorylation and accumulation of Tau induced synapse dysfunction and loss that was sufficient to induce cognitive impairments without significant widespread neuronal atrophy.^12^ This suggests that pTau induced a casual pathophysiology in synapse without necessarily disrupting the whole neuron.

One possibility that could drive the pathophysiology is the impact of pTau upon AMPAR turnover and/or trafficking.^5,7^ Our previous study illustrated the importance of specific Tau phosphorylation sites (396/404; Tau-PHF1E) in mediating LTD in Shaffer collateral-CA1 synaptic plasticity of the hippocampus.^3,7^ Despite these insights, the causal mechanism of aberrant pTau induced hippocampal synapse dysfunction remains elusive. Specifically, a knowledge gap exists in understanding how pTau induced pathophysiology progresses, especially considering that synapse dysfunction can occur without total neuronal atrophy. This opens the possibility that the small dendritic regions could undergo synapse dysfunction and underpin wider cognitive impairments. Therefore, it is of interest to understand the role of pTau in synapses, which may be a fundamental pathophysiology and causes synapse dysfunction and leads to cognitive deficits.

The hippocampus is a key structure responsible for cognitive processing, and a brain area that exhibits significant pathology during neurodegeneration. Specifically, during the early stages of the disease, synapse loss and dysfunction are observed in the hippocampus.^13,14^ The hippocampus itself exhibits a strong spatial organization, with well-defined regions and circuitry. However, how this circuitry impacts the progression of pathophysiology is not fully understood. For example, in various AD/FTLD models (APP.V7171 and TauP301L), a significant impairment in LTP is observed in the CA1 region while LTP and synapse function remained normal in the CA3 region, despite the transgene being expressed in neurons at both regions.^15^ Thereby, illustrating that the underlying neuronal function, and or circuitry, might exacerbate or reduce pathophysiology (i.e. specific synapses may be more vulnerable). Therefore, here we further explored this concept and examined if specific regions of the same dendrite exhibit a differential pathophysiology in response to Tau-PHF1E expression. Specifically, we focused on the stratum radiatum located dendrites of the CA1 neurons, as these dendrites receive inputs from CA3 neurons essential for learning and memory.^16^ Interestingly, these stratum radiatum dendrites are not uniform and exhibit structural differences in the organization of inputs and function, which could result in a different expression of pathophysiology. For example, differences in CA3–CA1 synapse connectivity exist between proximal and distal regions of the dendrite, with a greater number of multiple synapses at distal dendritic sites.^17,18^ Furthermore, these distal dendritic regions exhibit a higher presynaptic release probability.^18^ This suggests that the distal dendritic region of the stratum radiatum may be of high importance for synaptic transmission and learning and memory. In this regard, it is of interest whether Tau-PHF1E-mediated pathophysiology, which is known to induce synapse weakening,^3,7^ exhibits a uniform or spatially segmented progression of pathophysiology, due to differences in synaptic activity and/or architecture of synapse within the same stratum radiatum dendrite of CA1 hippocampal neuron.

Here, we illustrate that Tau-PHF1E, a human tau phosphomimic construct, exhibited a spatial distribution of pathophysiology, with the distal dendric regions of stratum radiatum exhibiting dysfunction while the proximal region of the same dendrites remained unaffected. Furthermore, we observed that these deficits were dependent upon the presence of a key tau-interacting protein; protein kinase C and casein kinase substrate in neurons protein 1 (PACSIN1). Collectively, suggesting that Tau-PHF1E can induce distal-region specific pathophysiology in stratum radiatum CA1–CA3 network, in the hippocampus.

## Materials and methods

### Animals

All procedures involving animals were carried out in accordance with the UK Animals Scientific Procedures Act, 1986. Male postnatal Day 6–7 Wistar rats (Charles River, UK) were used to prepare organotypic hippocampal slice cultures. All animals were maintained in standard enriched housing and a 12 h light–dark cycle. All animal experiments were given ethical approval by the ethics committee of King’s College London (protocol reference U214).

### Organotypic slice culture model

Hippocampal organotypic slices were cultured as previously described (see Kimura *et al*.^2^ and Regan *et al*.^7^). Briefly, rat pups were decapitated and the brain rapidly removed and placed into ice-cold dissecting medium containing: sucrose 239 mM, NaHCO_3_ 26 mM, D-glucose 11 mM, MgCl_2_ 5 mM, KCL 2.5 mM, NAH_2_PO_4_ 1 mM and CaCl_2_ 1 mM. Hippocampi were extracted and transverse slices (350 μm) made with a tissue chopper. Individual slices were placed upon a sterile, semi-porous membrane and stored at the interface between air and culture medium containing 78.8% minimum essential medium with L-glutamine, 20% heat inactivated horse serum, HEPES 30 mM, D-glucose 26 mM, NaHCO_3_ 5.8 mM, CaCl_2_ 2 mM, MgSO_4_ 2 mM, ascorbic acid 70 μm and 1 μg/mL^−1^ insulin (pH adjusted to 7.3 and osmolarity adjusted to 320–330 mOsm kg^−1^), inside a humidified incubator at 35°C with a 5% CO_2_ enriched environment.

### Biolistic transfection

Organotypic slices were biolistically transfected at DIV 3–4 using the Helios Gene Gun system (Bio-Rad), as previously described.^2,7^ Following transfection, slices were returned to the incubator until experiments were performed at DAT 3–5. cDNA for full-length pseudophosphorylation mutants of human full-length tau (2N4R) were expressed in a pCI-neo vector and co-transfected with rat tau shRNA. Specifically, a mixture of four different tau shRNA constructs (OriGene Technologies, USA) was used to silence tau as previously described.^2^ The human full-length Tau (2N4R) contained point mutations to induce pseudophosphorylation (mutated to glutamine; Tau-PHF1E) or phosphonul (mutated to alanine; Tau-PHF1A) at serine residues 396 and 404, respectively. When required, additional functional and structural markers were co-expressed with Tau; structural markers—tdTomato-C1 (Addgene; 54653), Venus and endogenous PSD95 marker.^19^ GCaMP7 was expressed by pGP-CMV-jGCaMP7s, which was a gift from Douglas Kim & GENIE Project (Addgene plasmid 104463).^20^ To visualize CaMKII, we utilized pCAG-mEGFP-CaMKIIa that was a gift from Ryohei Yasuda (Addgene plasmid 127389).^21^ For specific experiments, PACSIN1 was knocked down by shRNA (GCAGCTACATTCACGTCTATC) expressed under the control of the pENTR/U6 vector, designed in-house, and previously validated.^7^

### Multiphoton imaging

Organotypic cultured slices were transferred to an immersion chamber containing and imaged with a Scientifica hyperscope equipped multiphoton Coherent Chameleon laser and a Nikon 25× lens (CFI75, 25×, 1.1 NA). All regions of interest were acquired at 17.7 px/μm. Fluorophores in the green range were excited at 920 or 950 nm when acquired in combination with red fluorophores.

### Single spine structural plasticity

Multiphoton galvo scanning was utilized to acquire baseline Z-stacks (0.5 μm step size, 10× average). Images were acquired for 15 min at 5-min intervals before single dendritic spines were activated by uncaging of MNI-Glutamate (5 mM, HelloBio, UK) at 720 nm (20 ms, 2 Hz, 99 repeats). Subsequently, post-stimulation images were acquired at 5-min intervals for 30 min. Images were averaged offline and spine plasticity calculated as an increase in spine area (calculated from normalized baseline values). To assess if significant plasticity had occurred, unpaired *t*-test comparing against neighbouring unstimulated spines was performed. All spines were normalized to the baseline period, and spines from each transfection and stimulation group were combined to perform one-tailed *t*-test against unstimulated spine from the same region of interest (ROI). Comparison across transfection groups was performed by one-way ANOVA with *post hoc* Tukey analysis at the 30 min post-stimulation time point.

### CaMKII-GFP recruitment assay

A similar approach to the structural plasticity assay described above was utilized here. Multiphoton galvo scanning was utilized to acquire baseline Z-stacks (0.5 μm step size, 10× average). Images were acquired for 15 min at 5-min intervals before single dendritic spines were activated by uncaging of MNI-Glutamate (5 mM, HelloBio, UK) at 720 nm (20 ms, 2 Hz, 99 repeats). Subsequently, post-stimulation images were acquired at 5-min intervals for 30 min. Images were averaged offline the td-tomato and CaMKII-GFP channels separated. We use the ration of green/red (G/R) fluorescence as a measure of CaMKII concentration, as previously described in Zhang *et al.* (2008).^21,22^ Briefly for each spine, the G/R ration was calculated and normalized to the G/R ration in the dendrite; thereby, compensating for difference in the relative expression level of the two constructs across neurons. To allow for comparison between experimental groups, the CaMKII concentration following glutamate uncaging was normalized to the baseline CaMKII concentration.

### Fluorescent recovery after photobleaching (FRAP) assays

Baseline single z-plane images were acquired for 30 s at 2 Hz. The selected region of interest was the bleached with 950 nm laser following the baseline recording and the subsequent recovery monitored for 5 min. Images were averaged with a factor of two and normalized to the baseline fluorescent intensity.

### GCaMP7 imaging

GCaMP7 transfected neurons (±TauPHF1E) were imaged on the multiphoton microscope utilizing resonant scanning (30 Hz) at a single z-focus containing distally located dendritic spines. Images were acquired for 4 min. Following acquisition, data were processed by grouped average z-stack to obtain a 2 Hz file. A max intensity projection was created and ROIs created within the boundary of dendritic spines. The mean intensity value for the ROI was acquired from each frame a peak detected. Event frequency and amplitude were determined across the entire 4 min recording. To account for variation between neurons, event amplitude for each neuron was normalized to its individual baseline, and as such is expressed as a per cent value.

### Spinning disk confocal imaging

Organotypic cultured slices were transferred to an immersion chamber containing and imaged on a Nikon upright spinning disk microscope equipped with a 100× Nikon Lens (100×, 1.10 NA). The endogenous PSD-95 puncta were imaged with Z-stacks (4× average, 0.4 μm step size) and the puncta density and area analysed offline (Fiji).

### Statistical analysis

Statistical analysis was performed using GraphPad Prism software or SPPS as appropriate. For glutamate uncaging assays, the *n*-value refers to the number of individual dendritic spines, consistent with the standard practise in the field for this experimental paradigm. In each experiment, a single neuron/brain slice was used, and one pair of dendritic spines, one from the proximal and one from the distal region, were randomly selected. As only one neuron (i.e. pair of spines) was analysed per brain slice, each observation can be reasonable considered to be statistically independent. As such, the dendritic spine is treated as the experimental until for the glutamate uncaging assay. However, to avoid pseudo-replication, only one set of paired spines (proximal and distal) was examined per neuron, and only one neuron per brain slice and the total number of individual animals is listed in the text. All other datasets were analysed via a mixed effects model by grouping neurons/brain slices from individual animals into ‘nests’ and performing either independent two-tailed nested *t*-tests or nested one-way ANOVA, followed by *post hoc* test Tukey analysis to correct for the multiple comparison. Sample sizes and animal numbers are described in figure legends and were based on preliminary findings of the minimum number of samples that are required to detect statistically significant differences in the group means, with an observed power of at least 0.8. All data are presented as mean ± SEM.

## Results

### Tau-PHF1E impaired synaptic plasticity only at the distal region of stratum radiatum

**Figure 1:**
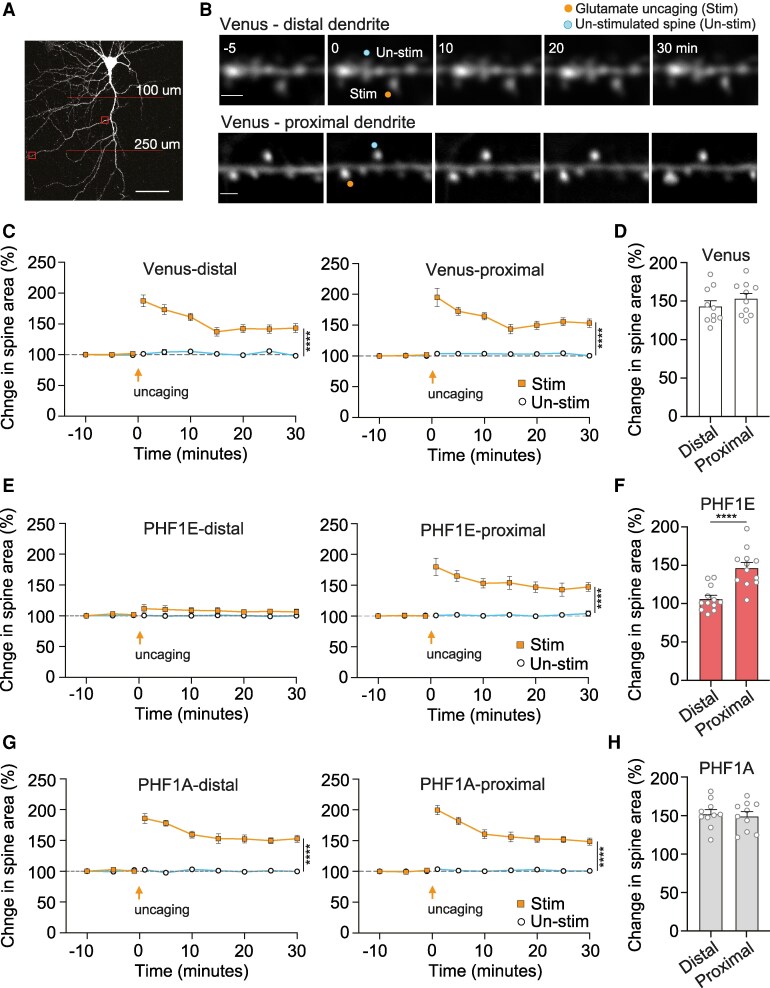
**TauPHF1E induced a distal specific inhibition of single spine plasticity**. (**A**) Example Venus transfected neuron (1× digital magnification) illustrating an example proximal and distal dendritic ROI selection (box) and the range at which we selected the secondary dendritic branch (100–250 μm from soma) to ensure that they are within the stratum radiatum. Scale bar 100 μM. (**B**) Representative time course images of proximal and distal located dendritic spines of the neurons prior to and following single spine glutamate uncaging stimulated (orange circle) and unstimulated spines (blue circle) in neurons transfected with Venus. Scale bar 1 μM. (**C**, **E**, **G**) Time-course graphs illustrating the average change in spine area (normalized to baseline) for distal (left) and proximal (right) stimulated (square) and unstimulated (circle) spines for Venus (**C**), TauPHF1E (**E**) and TauPHF1A (**G**). (**D**, **F**, **H**) Histogram illustrating the average change in spine area at 30 min post-stimulation for stimulated distal and proximal spines. ****P < 0.0001 unpaired *t*-test between unstimulated and stimulated spines at 30 min post-stimulation time point, or between proximal and distal stimulated spines at 30 min time point. All points indicate individual dendritic spines, only a single set of recordings was obtained per neuron. Error bars indicate SEM.

Structural and functional plasticity in dendritic spines is correlated with circuit plasticity observed during learning and memory.^23,24^ As the stratum radiatum synapse circuitry has distinct connectivity,^17,18^ therefore, it is of interest to examine if different regions of the stratum radiatum dendrite exhibit a different vulnerability to pTau pathophysiology. Here, we examined two distinct regions of interest, the first within the immediate proximal regions close to the dendritic branching point and the second at the distal regions of the same secondary apical stratum radiatum dendrite (Fig. 1A). The neurons were biolistically transfected with human Tau phosphomimic (Tau-PHF1E) or tau phosphonul (Tau-PHF1A) and a structural marker, and we analysed activity-dependent single dendritic spine plasticity in the proximal and distal regions that were stimulated via two-photon glutamate uncaging.

Distally, glutamate uncaging induced structural plasticity in both Venus transfected control neurons (143.2 ± 7.3, *n* = 10 neurons, six rats, *t*(18) = 5.693, *P* < 0.0001 unpaired *t*-test; Fig. 1B–D) and Tau-PHF1A (152.8 ± 5.7, *n* = 10 neurons, five rats, *t*(18) = 9.437, *P* < 0.0001 unpaired *t*-test; Fig. 1G and H) when compared with unstimulated neighbouring spines. However, for neurons expressing Tau-PHF1E, distally located spines did not show any increase in spine area following stimulation via glutamate uncaging when compared with stimulated neighbouring spines on the same dendrite (106.4 ± 4.6, *n* = 12 neurons, seven rats, *P* = *t*(22) = 0.9952, *P* = 0.3302 unpaired *t*-test, Fig. 1E and F). Comparison of the distally located stimulated spines at 30 min post-stimulation between transfection groups indicated a significant effect of Tau on structural plasticity (*F*(2,29) = 18.52, *P* < 0.0001, one-way ANOVA). Venus transfected control and Tau-PHF1A stimulated distal spines exhibited a similar level of structural plasticity (*P* = 0.5043, Tukey *post hoc* analysis). Interestingly, Tau-PHF1E lack of structural plasticity was significantly impaired when compared to Venus (*P* = 0.003, Tukey *post hoc* analysis) and Tau-PHF1A (*P* < 0.0001, Tukey *post hoc* analysis). Conversely, in the proximal dendritic region, all neurons, Venus (control), Tau-PHF1A and Tau-PHF1E, exhibited an increase in the area of the stimulated spine that remained increased 30 min following stimulation (Fig. 1). Thereby suggesting that human Tau-PHF1E dysregulated synaptic plasticity only in the distal region of the dendrite.

### Tau-PHF1E induced a reduction of spine density only in the distal region of stratum radiatum

**Figure 2:**
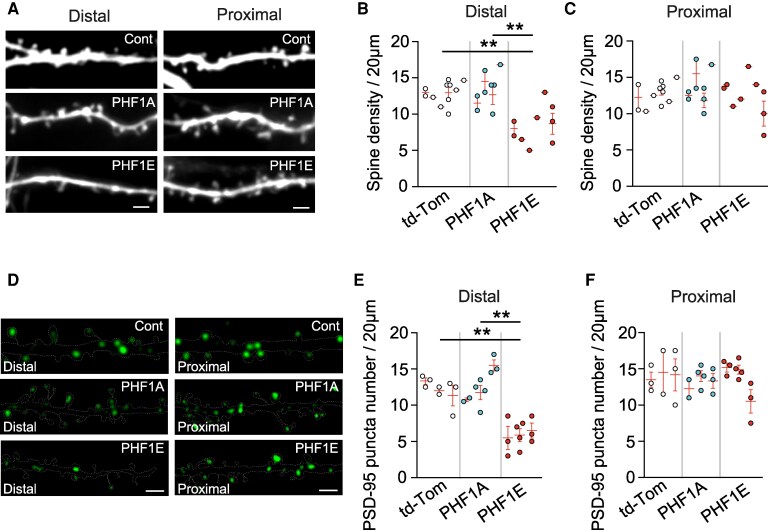
**TauPHF1E induced a distal specific reduction in spine and PSD-95 puncta density in the stratum radiatum of CA1 neuron**. (**A**) Representative image of spine density in the distal and proximal dendritic regions of apical secondary dendrite for control (td-tomato), PHF1A + td-tomato and PHF1E + td-tomato transfected neurons. (**B**, **C**) Average spine density (per 20 μm) for control (td-tomato, *n* = 11 neurons, six rats; white circle), PHF1A (*n* = 8 neurons, four rats; blue circle) and PHF1E (*n* = 9 neurons, six rats; red circle) transfected neurons in the distal (**B**) and proximal (**C**) regions of the stratum radiatum of CA1 neuron. (**D**) Representative images PSD-95 puncta in distal and proximal dendrite regions of the same apical secondary dendrite for control (td-tomato), PHF1A and PHF1E transfected neurons. Dotted white line illustrates dendrite boundary obtained from td-tomato channel. (**E**, **F**) Histogram illustrating PSD-95 puncta number (per 20 μm) for control (td-tomato, *n* = 8 neurons, three rats; white circle), PHF1A (*n* = 9 neurons, three rats; blue circle) and PHF1E (*n* = 10 neurons, three rats; red circle) transfected neurons in the distal (**E**) and proximal (**F**) regions of the stratum radiatum of CA1 neuron. Each point illustrated on the graph is an individual neuron. Neurons from the same rat are grouped into nests with the mean and SEM for each rat illustrated via the red bar. Scale bar 2 μM. **P < 0.01, *post hoc* Tukey analysis (nested one-way ANOVA); ***P < 0.001, *post hoc* Tukey analysis (nested one-way ANOVA).

Subsequently, we aimed to examine if the expression of Tau-PHF1E could affect the overall spine density in both the proximal and distal regions. We illustrated an effect of Tau-PHF1E in the distal dendritic region on spine density (*F*(2,13) = 14.4, *P* = 0.0005, nested one-way ANOVA). Specifically, Tau-PHF1E (8.44 ± 0.86 spines/20 μm, *n* = 9 neurons, six rats) caused a reduction of spine density when compared to both control (12.92 ± 0.46 spines/20 μm, *n* = 11 neurons, six rats, *P* = 0.0013, *post hoc* Tukey analysis) and Tau-PHF1A (13.35 ± 0.85 spines/20 μm, *n* = 8 neurons, four rats, *P* = 0.0011, *post hoc* Tukey) transfected neurons (Fig. 2A and B). Conversely, in the proximal dendritic region, Tau-PHF1E (12.33 ± 0.92 spines/20 μm, *n* = 9 neurons, six rats) did not exhibit any reduction in spine density (*F*(2,13) = 0.5553, *P* = 0.5870, nested one-way ANOVA) when compared to either control (12.70 ± 0.49 spines/20 μm, *n* = 11 neurons, six rats, *P* = 0.9993, *post hoc* Tukey analysis) or Tau-PHF1A (13.53 ± 0.88 spines/20 μm, *n* = 8 neurons, four rats, *P* = 0.6443) transfected neurons (Fig. 2A and C).

To further examine the effect of Tau-PHF1E on excitatory synapse structure, we examined the expression of a key excitatory postsynaptic scaffolding protein; postsynaptic density 95 (PSD-95). To achieve this, we utilized a plasmid to tag endogenous PSD-95,^19^ and monitored its expression in the distal and proximal regions of the same dendrite (Fig. 2D–F). Tau-PHF1E induced a reduction of PSD-95 puncta number only in the distal region of the dendrite in the stratum radiatum (*F*(2,6) = 18.32, *P* = 0.0028, nested one-way ANOVA). Specifically, Tau-PHF1E when compared with the Tau-PHF1A (*P* = 0.0039, *post hoc* Tukey analysis) or td-Tomato (control; *P* = 0.0061, *post hoc* Tukey analysis) transfected neurons (Fig. 2E and F). Collectively, suggesting that in addition to impairing the function of the spines that remain in the dendritic region, Tau-PHF1E additionally induced the loss of spines in the distal region that will eliminate essential synaptic inputs.

### Tau-PHF1E impaired calcium transients and CaMKII recruitment to distal dendritic spines

**Figure 3:**
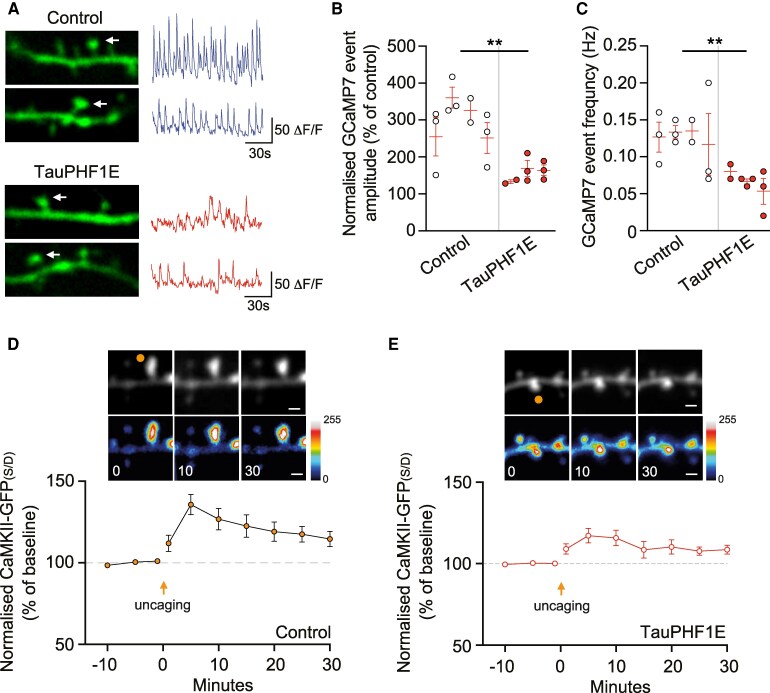
**TauPHF1E dysregulates dendritic spine calcium transients and CaMKII activity**. (**A**) Representative image and corresponding trace illustrating the GCaMP7 signal in a distal dendritic spine for control (*top*) and TauPHF1E (*bottom*) transfected neurons. (**B**, **C**) When compared to control (*n* = 11 neurons, four rats), TauPHF1E (*n* = 8 neurons, three rats) induced a decrease in normalized GCaMP7 transient amplitude (**B**) and frequency (**C**). For **C** and **D**, each point illustrated is an individual neuron. Neurons from the same rat are grouped into nests with the mean and SEM for each rat illustrated via the red bar. (**D**) Representative multiphoton time course images illustrating structure (TdTomato) and CaMKII-GFP localization for control neurons. Below, the mean normalized CaMKII-GFP concentration is illustrated against time for control distal dendrites (*n* = 7 neurons, three rats). (**E**) Representative multiphoton time course images illustrating structure (TdTomato) and CaMKII-GFP localization for TauPHF1E transfected neurons. Below, the mean normalized CaMKII-GFP concentration is illustrated against time for TauPHF1E transfected distal dendrites (*n* = 6 neurons, three rats). For glutamate uncaging assays, each dendritic spine is considered a replicate with only one spine being taken per neurons/brain slice. Scale bar 1 μM. **P < 0.01, nested *t*-test.

We next aimed to examine differences between control and TauPHF1E distal spines to determine how structural LTP is impaired. Initially, we transfected CA1 neurons with the genetically encoded calcium indicator (GCaMP7) and measured spontaneous calcium transients in the distal spines (Fig. 3A). We observed that TauPHF1E induced a significant reduction in the normalized amplitude (*t*(5) = 4.039, *P* = 0.0099, nested *t*-test; Fig. 3B) and frequency (*t*(5) = 4.187, *P* = 0.0086, nested *t*-test; Fig. 3C) of the GCaMP7 events. Thereby suggesting that TauPHF1E impairs the overall calcium dynamics within the spines. Subsequently, we analysed CaMKII concentration within the distal dendritic spines under baseline and following the LTP glutamate uncaging paradigm. We observed that single spine glutamate uncaging induced a robust increase in CaMKII in control distal spines by 10 min (Fig. 3D) that remained increased at 30 min (*t*(12) = 2.814, *P* = 0.156, *n* = 7). Conversely, in TauPHF1E transfected neurons, there was relatively less robust increase in CaMKII (*t*(11) = 2.261, *P* = 0.0450, *n* = 13 control 30 min time point versus TauPHF1E 30 min time point) that did not remain elevated at 30 min (*t*(10) = 2.198, *P* = 0.0562, *n* = 6, Fig. 3E). Collectively, indicating TauPHF1E induced dysregulation of calcium and CaMKII activity in distal spines.

### Tau-PHF1E induced a reduction of PSD-95 mobility only in the distal region of stratum radiatum

**Figure 4:**
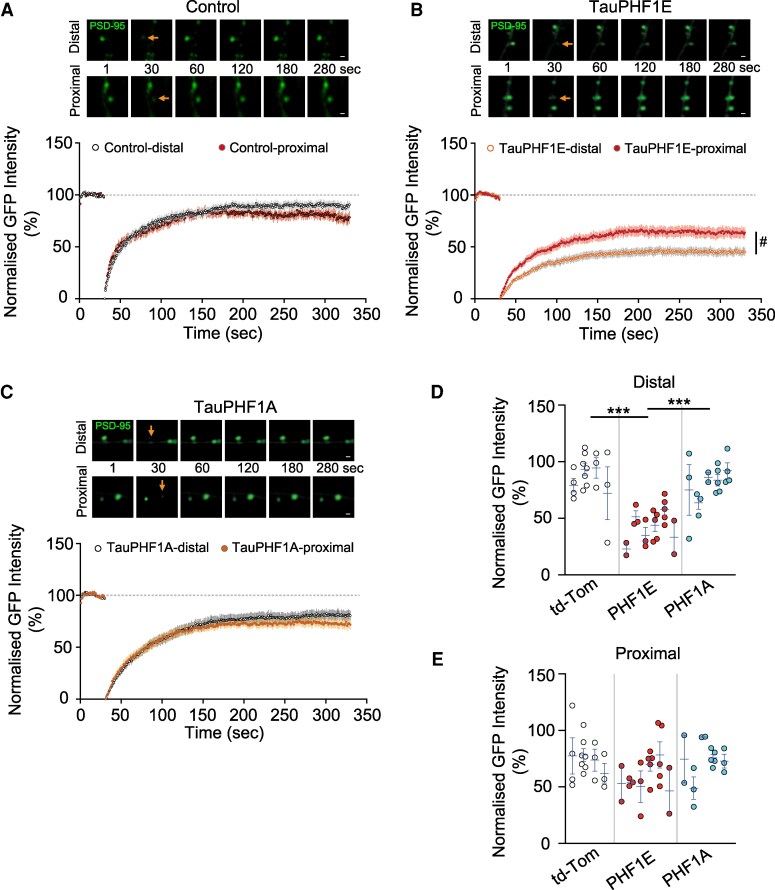
**TauPHF1E impaired postsynaptic density 95 (PSD-95) protein mobility in the distal region of stratum radiatum of CA1 neuron**. (**A–C**) Top representative time course image of PSD-95 puncta prior to and post-bleaching (arrow) for control (**A**), PHF1E (**B**) and PHF1A (**C**) transfected neurons in the proximal and distal dendritic regions. Below each representative time course is the corresponding averaged normalized fluorescent recovery after photobleaching (FRAP) of PSD-95 puncta located for distal and proximal regions of TauPHF1E and TauPHF1A transfected neurons. (**D**) Summary bar chart illustrating the normalized PSD-95 puncta intensity following recovery for control (*n* = 17 neurons, four rats), TauPHF1E (*n* = 20 neurons, six rats) and TauPHF1A (*n* = 17 neurons distal, five rats) transfected neurons. (**E**) Summary bar chart illustrating the normalized PSD-95 puncta intensity following recovery for proximal regions of control (*n* = 17 neurons, four rats), TauPHF1E (*n* = 20 neurons, six rats) and TauPHF1A (*n* = 15 neurons, five rats) transfected neurons. (**A–C**) Each point is the mean ± SEM for the time point illustrated. (**D**, **E**) Each point illustrated on the graph is an individual neuron. Neurons from the same rat are grouped into nests with the mean and SEM for each rat illustrated via the blue bar. Scale bar 1 μM. ^#^*P* < 0.05, unpaired nested *t*-test; ***P < 0.001, nested one-way ANOVA *post hoc* Tukey. Error bars indicate SEM.

Since Tau-PHF1E caused a reduction of PSD-95 in the distal dendritic region, it was of interest whether the overexpression of Tau-PHF1E could affect the dynamics of PSD-95 mobility. The postsynaptic density is a mobile structure that constantly shuttles new PSD-95 protein in and out of the synaptic domain.^25,26^ Furthermore, these dynamic properties have been illustrated to be required for both LTP and LTD.^27^ Interestingly, Tau is known to directly interact with PSD-95^28^ and pTau has been shown to alter this interaction.^29^ We aimed to determine if pTau induced an alteration in PSD-95 dynamics by fluorescent recovery after photobleaching (FRAP). When each group was considered separately, only Tau-PHF1E exhibited a difference in PSD-95 recovery between proximal and distal (*t*(10) = 2.247, *P* = 0.0484, nested *t*-test, *n* = 21 neurons, six rats, Fig. 4B). Whereas Tau-PHF1A (*t*(8) = 0.9698, *P* = 0.3606, nested *t*-test, *n* = 15 neurons, five rats, Fig. 4C) and control (mCerulean; *t*(6) = 1.759, *P* = 0.1291, nested *t*-test, *n* = 17 neurons, four rats, Fig. 4A) had similar levels of recovery between proximal and distal regions. Subsequently, comparing across transfection groups, we observe that the FRAP recovery of PSD-95 was similar between proximal regions of control (mCerulean), Tau-PHF1E and Tau-PHF1A (*F*(2,12) = 1.862, *P* = 0.1976, nested one-way ANOVA, Fig. 4E). Conversely, in the distal regions of the same dendrites, we observed a significant effect of transfection (*F*(2,12) = 21.85, *P* < 0.0001, nested one-way ANOVA, Fig. 4D). Specifically, in the distal region, Tau-PHF1E exhibited a lower FRAP recovery that control (mCerulean *P* = 0.0002, *post hoc* Tukey analysis) and Tau-PHF1A (*P* = 0.0005, *post hoc* Tukey analysis). This suggests that Tau-PHF1E can cause the pathophysiology with the distal dendritic region predominantly exhibiting impaired PSD-95 mobility and/or expression.

### Tau-PHF1E induced distal pathophysiology is dependent upon the altered pTau:PACSIN1 interaction

**Figure 5:**
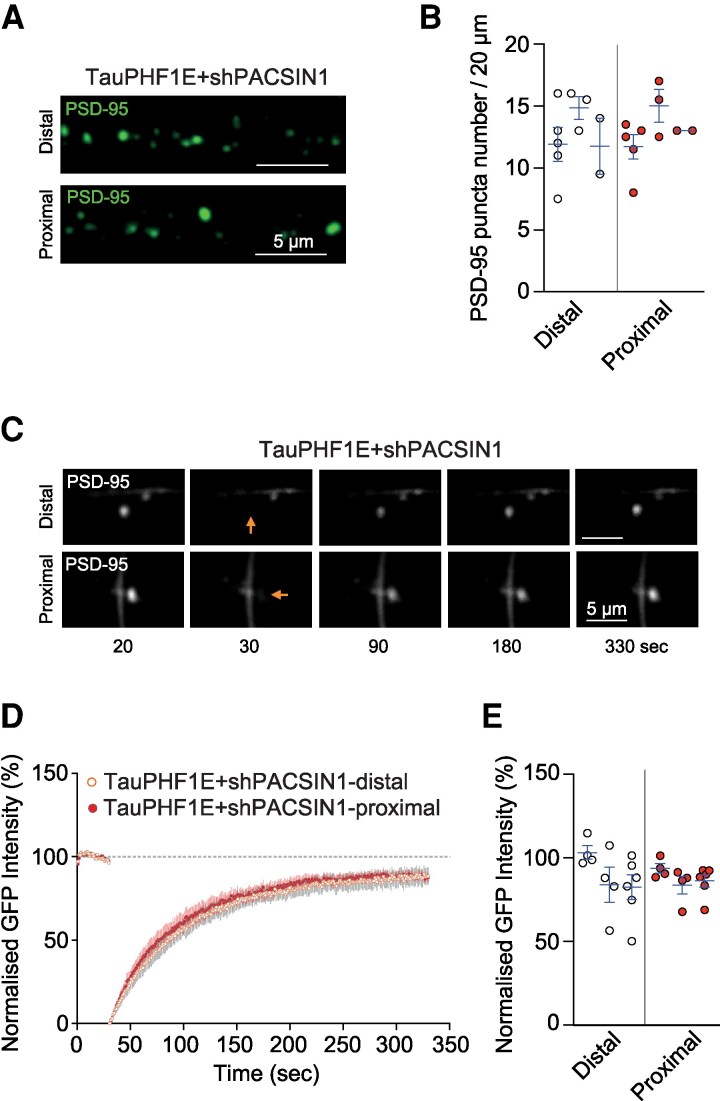
**Knock-down of PACSIN (shPACSIN) rescued the PHF1E induced reduction in PSD-95 puncta and FRAP recovery in distal dendritic spines**. (**A**) Representative image illustrating endogenous PSD-95-GFP puncta in the distal (*top*) and proximal (*bottom*) regions of PHF1E + shPACSIN1 transfected neurons. (**B**) Nested bar charts illustrating analysed PSD-95 puncta number between the distal and proximal regions of PHF1E + shPACSIN transfected neurons (*n* = 10 neurons, three rats). (**C**) Representative fluorescent recovery after photobleaching time course images of endogenous PSD-95-GFP intensity prior to and post-bleaching (arrow) in distal (*top*) and proximal (*bottom*) dendritic regions. (**D**) Normalized, averaged, endogenous PSD-95-GFP intensity for photobleached regions of interest at the distal (orange open circle, *n* = 14 neurons, three rats) and proximal (red closed circle, *n* = 14 neurons, three rats) of TauPHF1E + shPACSIN1 transfected neurons. (**E**) Nested histogram illustrating the normalized GFP intensity recovery in distal and proximal regions (*n* = 14 neurons, three rats). For all graphs, each point illustrated an individual neuron. Neurons from the same rat are grouped into nests with the mean and SEM for each rat illustrated via the blue SEM bar. Please see Supplementary Fig. 1 for nested analysis of PSD-95 puncta density and FRAP comparing multiple groups.

So far, our study suggests that synaptic dysfunction is dependently or independently coupled with a combination of pathophysiology mechanisms underpinned by the pTau. As we have previously observed,^7^ the pTau-mediated synapse weakening is associated with the tau protein interactome, specifically, protein kinase C and casein kinase substrate in neurons protein 1 (PACSIN1),^7^ a key molecule that links tau to the AMPAR endocytosis machinery (e.g. PICK1) and structural components of the spine (e.g. WASP and ARP2/3).^30^ Therefore, we wondered whether pTau induced alterations to PACSIN1 could potentially contribute to the distal pathophysiology. To examine this, we co-expressed shRNA PACSIN1 in addition to the tau constructs (Tau-PHF1E and Tau-PHF1A) and examined PSD-95 expression and mobility and single spine plasticity. We have previously confirmed that the shPACSIN1 construct induced a reduction in PACSIN1 expression.^7^ Initially, we quantified the number of excitatory synapses, by quantifying PSD-95-GFP puncta, and knock-down of PACSIN1 (shPACSIN1) rescued the previously observed Tau-PHF1E induced deficit in the distal region of the dendrite (*t*(4) = 0.1931, *P* = 0.8563, nested *t*-test Fig. 5A and B). Additionally, the proximal and distal regions exhibited the same PSD-95 mobility (i.e. same rate of recovery following FRAP; *t*(4) = 0.2186, *P* = 0.8377, nested *t*-test; Fig. 5C–E, Supplementary Fig. 1). These assays suggest that the Tau-PHF1E induced reduction in the distal dendritic PSD-95 density and protein mobility was dependent upon PACSIN1.

**Figure 6:**
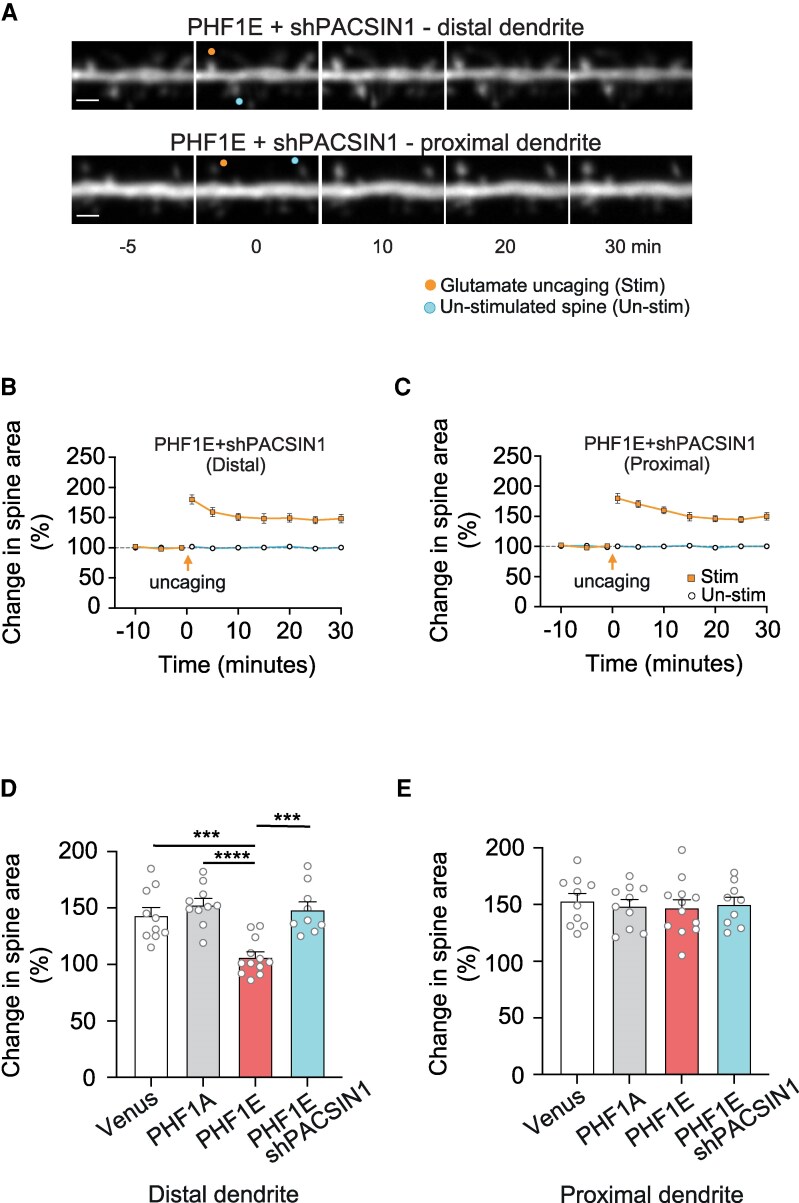
**Knock-down of PACSIN (shPACSIN) rescued the PHF1E induced impairment in single spine plasticity in distal dendritic spines**. (**A**) Representative time course images of TauPHF1E + shPACSIN1 transfected distal and proximal located dendritic spines of the neurons prior to and following single spine glutamate uncaging. (**B**, **C**) Graphs illustrating the average change in spine area (normalized to baseline) for distal (**B**) and proximal (**C**) stimulated and unstimulated spines of TauPHF1E + shPACSIN1 transfected neurons. Scale bar 1 μM. (**D**, **E**) Bar charts illustrating the increase in spine are following glutamate uncaging for distal (**D**) and proximal (**E**) spines transfected with Venus (white bar; *n* = 10 neurons, six rats), TauPHF1A (grey bar; *n* = 10 neurons, 10 rats), TauPHF1E (red bar; *n* = 12 neurons, seven rats) and TauPHF1E + shPACSIN (blue bar; *n* = 9 neurons, four rats). Please note that in **D** and **E**, the data for Venus, PHF1A and PHF1E are represented and reanalysed from Fig. 1. ***P < 0.001, ****P < 0.0001, nested one-way ANOVA *post hoc* Tukey. Error bars indicate SEM.

We next aimed to determine if shPACSIN1 rescued the functional properties of the distal synapses by utilizing the two-photon glutamate uncaging induced spine structural plasticity. Interestingly, for Tau-PHF1E + shPACSIN1 transfected neurons, both the distally (148.2 ± 7.1, *n* = 9 spines, nine neurons, seven rats, *t*(16) = 6.419, *P* < 0.0001 unpaired *t*-test; Fig. 6A and B) and proximally (150.1 ± 5.1, *n* = 9 spines, nine neurons, seven rats, *t*(16) = 7.566, *P* < 0.0001 unpaired *t*-test; Fig. 6A and C) located spines now underwent a significant structural plasticity. Subsequently, we compared the structural plasticity of TauPHF1E + shPACSIN1 with those groups previously described in Fig. 1. Comparison between groups indicated a significant effect of Tau (*F*(3,37) = 13.19, *P* < 0.0001 one-way ANOVA) on the ability to undergo structural plasticity. Specifically, Tau-PHF1E + shPACSIN1 did not differ from control (*P* = 0.9434, *post hoc* Tukey) or TauPHF1A (*P* = 0.9565, *post hoc* Tukey) transfected neurons but were significantly different from TauPHF1E (*P* = 0.0001, *post hoc* Tukey) transfected neurons (Fig. 6D and E). Collectively, the data illustrate that shPACSIN1 can rescue the observed functional pathophysiology induced in the distal region by Tau-PHF1E.

## Discussion

Here, our study postulates that Tau-PHF1E induced different pathophysiology dependent upon the synapse location within the dendritic arbour of the CA1 neuron. Specifically, Tau-PHF1E dysregulated PSD-95 protein expression and/or mobility, and activity-dependent long-term synaptic plasticity in the distal dendritic region, but not the proximal region of the same secondary dendritic branch in the stratum radiatum of CA1 neuron. Collectively, these findings provide a new insight and postulate a novel spatial distribution of pTau pathophysiology with the distal dendritic regions exhibiting a weakening of the excitatory synapses and a higher vulnerability. Interestingly, a comparable segmentation of synapse weakening has previously been observed in senile dementia of Alzheimer’s type patients. Specifically, that granular cells distal regions exhibit synapse loss whereas proximal regions did not.^10^ The authors attributed this difference to the underlying circuitry and differing synaptic inputs between the two regions. Similarly, in our model, the neuroanatomical synapse-architecture of the CA1 neurons and the hippocampus produces distinct differences between distal and proximal regions of these dendrites, which may itself underpin the micro-subregional pathophysiology. Ultimately, a local dysregulation within a specific region of the CA1 neurons might be sufficient to induce significant phenotypes, such as cognitive impairments.^12,15^ Interestingly, the distal synapses that remained exhibited an impairment in PSD-95 mobility and single spine plasticity. A similar finding was illustrated in the hippocampal circuit, where ATR-X was observed to significantly impair distal LTP but only marginally impair proximal LTP.^11^

The association between PSD-95 mobility and plasticity is well known,^25,26^ as reductions in PSD-95 mobility have been shown to impair both LTP and LTD.^27^ Furthermore, Tau is known to interact with PSD-95^31^ and the phosphorylation of Tau results a significant reduction in Tau:PSD-95 interaction while also increasing AMPAR endocytosis in a PACSIN1:PICK1 dependent manner.^7^ Therefore, here we postulate that pTau causes a subregional synapse weakening by increasing AMPAR endocytosis-associated mechanism and impairing PSD-95 mobility in the distal dendritic regions.

The downstream molecular mechanism for structural plasticity and LTP requires the rise of intracellular calcium and activation of CaMKII that subsequently activates and recruits various small GTPases (e.g. RhoA, Rac1 and Cdc42) leading the promotion of actin polymerization and spine enlargement.^22,32-35^ Specifically, it has been illustrated that RhoA activation results in transient spine growth while Cdc42 activation contributes to the maintenance of spine enlargement.^35^ In TauPHF1E transfected distal dendritic spines, we did not observe any transient enlargement of structural plasticity, thereby suggesting that the initial activation of plasticity was impaired. As such, we chose to investigate the level of spontaneous calcium rise within the spines and the amount of CaMKII recruited upon glutamate uncaging. We observed a significant reduction in the amplitude and frequency of calcium in the distal dendritic spines, coupled with the previously observed reduced AMPAR function,^7^ potentially indicating weaker synaptic depolarization and reduced excitatory drive that could lead to less activation of CaMKII and downstream GTPases. Similarly, we performed glutamate uncaging and monitored the concentration of CaMKII in distal dendritic spines before and after stimulation. We observed the classical increase in CaMKII localization for control neurons, but this was reduced in TauPHF1E transfected cells. Collectively suggesting a dysfunction in calcium signalling and leads to weakening CaMKII activity following single spine stimulation by TauPHF1E.

Furthermore, we identify that PACSIN1, a molecule known to play a key role in AMPAR endocytosis,^30^ neuronal cytoskeleton maintenance^36^ and TauPHF1E induced synapse weakening,^7^ or its downstream signalling, plays a key role in the observed distal synapse weakening. A key unknown factor is the distribution of PACSIN1 under endogenous and pathophysiological conditions. Subsequently, there is also a possibility that the interaction of Tau and PACSIN1 could show a distance dependent distribution that could then lead to the observed differences in pathophysiology. Additionally, PACSIN1 is known to directly interact with neural Wiskott–Aldrich syndrome protein (N-WASP).^36,37^ Importantly, the N-WASP/ARP2 pathway mediated the branching of actin that is proposed to be a mechanism for dendritic spine head enlargement and maturation.^38^ As such, the change in the Tau:PACSIN1 interaction induced by Tau phosphorylation, specifically TauPHF1E in this study, could impair the WASP/ARP2 pathway in distal spines.

We have adopted the same model and overexpression of TauPHF1E as our previous study,^7^ and as such have found supportive findings such as TauPHF1E-mediated synapse weakening and a PACSIN1 dependence. Specifically, we previously observed that TauPHF1E induced a synapse weakening phenotype, in terms of morphology but not density.^7^ We believe that this was due to the lack of examining specific segmentation within the previous study. Additionally, we observed a weakening of the AMPAR-mediated excitatory postsynaptic potential (EPSC) that were evoked via Schaffer collateral stimulation and recorded at the soma, this could be explained by the loss of synapses in the distal region due as this area receives strong synaptic input from the Schaffer collateral. Interestingly, tau phosphorylation at residue 396 has been indicated to be one of the earliest events in Alzheimer’s disease.^39^

Here, we utilized an overexpression of exogenous full-length phosphomimic Tau (2N4R Tau-PHF1E), or appropriate controls protein that does not represent endogenous levels of the protein. Interestingly, all appropriate controls (e.g. Tau-PHF1A, the phosphonul construct) were also overexpressed, and no phenotype of pathophysiology was observed. Furthermore, the fact that an overexpression resulted in regional specific differences excludes a possibility of non-specific effect and strengthens the findings, as one would expect the overexpression to cause neuron wide dysfunction. This study used human TauPHF1E, which mimics phosphorylation only at the S396/404 sites, therefore we have not exhibited other Tau hyperphosphorylation site (e.g. phosphorylation at all sites). The TauPHF1E is not a ‘disease’ model such as TauP301L, but the main aim of this study was to address the biology that underpins pTau-mediated pathophysiology associated with synapse weakening.^3,7^ Therefore, in the future, it would be of great interest to examine if disease models of pTau (e.g. TauP301L) exhibit a similar phenotype or if this role of tau is more related to homoeostatic function.

The current study, however, cannot reveal why the distal dendritic synapses are more vulnerable. One possibility could arise from the different inherent properties that exist between the proximal and distal regions of the dendrite in CA1 neurons. Interestingly, the presynaptic neurotransmitter release probability has been shown to increase towards the distal dendritic region when compared with proximal region of the same dendrite.^18^ In addition, the proportion of AMPAR has been shown to increase with distance from the soma.^40^ Thereby, collectively suggesting a different synaptic input–output strength at the proximal and distal dendritic regions that may cause activity-dependent local changes in translational regulation and protein dynamics/turnover.^41,42^ Alternatively, a differential expression of pTau and/or PACSIN1 within the dendrite could drive pathophysiology in specific regions. Additionally, pTau can induce microtubule instability and disrupted dendritic transport, which may in turn give rise to a distance dependent pathophysiology (e.g. reduced transport to the distal regions). However, how reducing PACSIN1 (shPACSIN1) could rescue the pathophysiology if it is due to defective transport is not clear, making this option less likely. Additionally, we should consider the tau interactome and how this is altered following its phosphorylation, as this may reveal novel synapse weakening pathways. Finally, we need to address if this is a circuit specific (i.e. CA3–CA1) or neuronal specific (i.e. distance dependent in all neurons) phenotype, as previous pTau models have illustrated circuit specific pathophysiology.^15^

Synapse dysfunction and loss are widely acknowledged to be a common prodromal pathophysiological feature of multiple neurodegenerative diseases.^12,43,44^ Crucially, synapse loss is correlated to cognitive decline in Alzheimer’s disease patients, thereby illustrating their importance and the potential causative role of synapse function in driving the cognitive symptoms of Alzheimer’s disease.^13,43,45,46^ Since synapse connectivity and coherent functionality of the CA1–CA3 are fundamental for the cellular and molecular mechanism of learning and memory,^47^ we postulate that the distal specific deficit within the stratum radiatum could underpin a significant synapse dysfunction without comprehensive dendritic or neuronal network atrophy, and may result in cognitive dysfunction observed in tauopathy and Alzheimer’s disease. Therefore, it will be of interest whether there is a different propagation of pathophysiology in association with the activity-dependent and the input–output properties of neural circuit in the future.

## Supplementary Material

fcaf234_Supplementary_Data

## Data Availability

No code/scripts were developed for the analysis of these data in this manuscript. All software and code utilized are detailed in the ‘Materials and methods’ and are available from the developers/manufactures websites (e.g. ScanImage and MBF Bioscience). All data are available from the corresponding author upon request.
