# Supplementary material for: Micro-sub regional synapse weakening by mimicking the hyperphosphorylation of microtubule associated protein Tau in dendritic spines
Source: Brain Commun. 2025 Jun 11;7(3):fcaf234. doi: 10.1093/braincomms/fcaf234 (PMC12199761; doi:10.1093/braincomms/fcaf234)
Supplement: fcaf234_Supplementary_Data [file fcaf234_supplementary_data.pdf]

Supplementary Materials

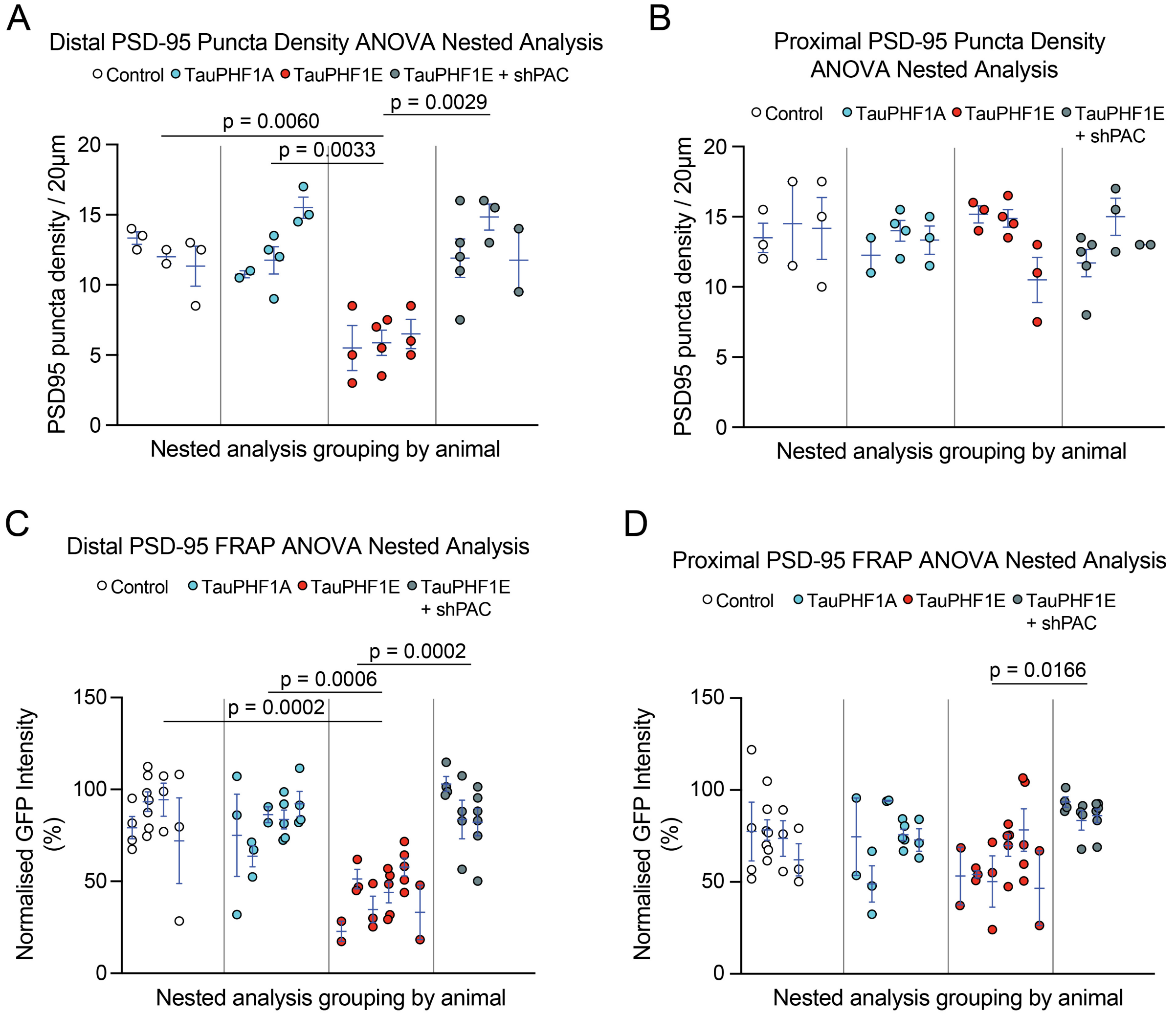

Supplementary Figure 1

A–B) Nested one-way ANOVA comparing PSD-95 puncta number between Control, Tau-PHF1E, Tau-PHF1A, and Tau-PHF1E + shPACSIN1 transfected neurons at **A)** distal and **B)** proximal regions. **C–D)** Nested one-way ANOVA comparing PSD-95 FRAP between Control, Tau-PHF1E, Tau-PHF1A, and Tau-PHF1E + shPACSIN1 transfected neurons at **C)** distal and **D)** proximal regions.

Note: Data for PSD-95 puncta number for Control, Tau-PHF1E, and Tau-PHF1A are replicated from Figure 2, and data for PSD-95 FRAP for Control, Tau-PHF1E, and Tau-PHF1A are replicated from Figure 4.

If a significant effect of transfection (i.e. Tau-PHF1E) was observed in the nested one-way ANOVA, post hoc Tukey analysis was performed, and any significant differences are illustrated on the figure with the stated *p*-value.

Data for Control, Tau-PHF1A, and Tau-PHF1E are also replicated from Figure 1. Each data point illustrated on the graph represents an individual neuron. Neurons from the same rat are grouped into nests, with the mean and s.e.m. for each rat illustrated by the blue bar.
